# Impact of the relationship between hemoglobin levels and renal interstitial fibrosis on long-term outcomes in type 2 diabetes with biopsy-proven diabetic nephropathy

**DOI:** 10.1186/s12882-021-02510-y

**Published:** 2021-09-25

**Authors:** Miho Shimizu, Kengo Furuichi, Shinji Kitajima, Tadashi Toyama, Megumi Oshima, Hisayuki Ogura, Koichi Sato, Shiori Nakagawa, Yuta Yamamura, Taro Miyagawa, Akinori Hara, Yasunori Iwata, Norihiko Sakai, Kiyoki Kitagawa, Mitsuhiro Yoshimura, Hitoshi Yokoyama, Takashi Wada

**Affiliations:** 1 Department of Nephrology and Laboratory Medicine, Graduate School of Medical Sciences, Kanazawa University, 13-1 Takara-machi, 920-8640 Kanazawa, Japan; 2 Health Service Center, Kanazawa University, Kanazawa, Japan; 3 Department of Nephrology, Kanazawa Medical University, Uchinada, Japan; 4 Department of Hygiene and Public Health, Graduate School of Medical Sciences, Kanazawa University, Kanazawa,, Japan; 5 Division of Internal Medicine, National Hospital Organization Kanazawa Medical Center, Kanazawa, Japan; 6 Division of Internal Medicine, Noto General Hospital, Nanao, Japan

**Keywords:** Diabetic kidney disease, Diabetic nephropathy, Hemoglobin, Interstitial fibrosis and tubular atrophy, Renal events, Mortality

## Abstract

**Background:**

Progression of renal anemia has been shown to be associated with advanced renal tubulointerstitial lesions. This retrospective study investigated the impact of lower hemoglobin (Hb) levels and renal interstitial fibrosis and tubular atrophy (IFTA) on long-term outcomes in type 2 diabetes with biopsy-proven diabetic nephropathy.

**Methods:**

A total of 233 patients were enrolled. The severity of IFTA was scored according to the classification by the Renal Pathology Society. Patients were stratified according to baseline Hb tertiles by IFTA status. The outcomes were the first occurrence of renal events (requirement for dialysis or 50 % decline in estimated glomerular filtration rate from baseline) and all-cause mortality.

**Results:**

At baseline, 151 patients had severe IFTA. There were no patients who have been received erythropoiesis-stimulating agents at the time of renal biopsy. The severity of IFTA was the independent pathological factor of lower Hb levels. During the mean follow-up period of 8.6 years (maximum, 32.4 years), 119 renal events and 42 deaths were observed. Compared with the combined influence of the highest tertile of Hb and mild IFTA, the risks of renal events were higher for the middle tertile and for the lowest tertile of Hb in severe IFTA, whereas the risk of renal events was higher for the lowest tertile of Hb in mild IFTA. The risk of mortality was higher for the lowest tertile of Hb only in severe IFTA. There were significant interactions of tertile of Hb and IFTA in renal events and mortality.

**Conclusions:**

Impacts of lower Hb levels on long-term outcomes of diabetic nephropathy were greater in severe IFTA than in mild IFTA.

## Background

Anemia in patients with diabetic nephropathy/diabetic kidney disease (DKD) develops at earlier stages than in patients with chronic kidney disease (CKD) from other causes [1, 2]. The average hemoglobin (Hb) concentration stratified by glomerular filtration rate (GFR) category is lower in patients with diabetic nephropathy/DKD than in those with other causes for CKD [1, 2]. Observational studies indicated that lower Hb levels in diabetic nephropathy/DKD were associated with adverse outcomes, including increased risks for progression of kidney disease, cardiovascular morbidity, and mortality [1, 2]. Nevertheless, most of these studies did not consider the renal pathology. Recently, pathological studies focusing on diabetic nephropathy [3], post-transplant nephropathy [4], and ANCA-associated renal vasculitis [5] demonstrated that the progression of renal anemia was associated with advanced renal tubulointerstitial lesions. Even though interstitial fibrosis and tubular atrophy (IFTA) is not specific in diabetic nephropathy [6], we and other investigators reported that the severity of IFTA was associated with renal events [7–14] and mortality [7, 9] in patients with type 2 diabetes and biopsy-proven diabetic nephropathy. Against this background, this long-term retrospective study investigated the impact of the relationship between Hb levels at the time of renal biopsy and IFTA on the risks for renal composite events and all-cause mortality in Japanese patients with type 2 diabetes and biopsy-proven diabetic nephropathy.

## Methods

### Subjects

We included 233 patients with type 2 diabetes and biopsy-proven diabetic nephropathy who were diagnosed at Kanazawa University Hospital or Kanazawa Medical Center between April 1985 and May 2019. The diagnosis of diabetes was determined by the criteria of the Japanese Diabetic Society [15] and/or medical history. Renal biopsy was performed with each patient’s consent to obtain precise diagnoses of kidney lesions. Diabetic nephropathy was diagnosed by confirming the typical histological features of diabetic nephropathy using light microscopy, immunofluorescence, and electron microscopy [7]. Patients with diabetic nephropathy complicated by other kidney diseases were excluded.

### Clinical examinations

The baseline was defined as the time of renal biopsy. Hemoglobin, age, gender, serum creatinine, estimated GFR (eGFR), 24-h urinary albumin excretion, 24-h urinary protein excretion, HbA1c, systolic blood pressure, diastolic blood pressure, total cholesterol, and body mass index were obtained as clinical covariates at baseline. eGFR was estimated using the equation of the Japanese Society of Nephrology [16].

### Pathological examinations

For light microscopic examinations, renal biopsy specimens were stained with periodic acid Schiff, periodic acid silver methenamine, hematoxylin–eosin, and Mallory–Azan. The severity of IFTA was evaluated semi-quantitatively according to the pathological classification by the Renal Pathology Society as follows: score 0, no IFTA; score 1, < 25 %; score 2, 25–50 %, and score 3, > 50 % [6–12, 17, 18]. The severity of diffuse lesion was graded from 0 to 4 by the method of Gellman et al. [7, 17–19]. Nodular lesion, exudative lesion, and mesangiolysis were scored as absent or present [7, 17, 18]. The severity of interstitial cell infiltration was graded from 0 to 2 according to the pathological classification by the Renal Pathology Society [6, 7, 17, 18]. The severity of arteriolar hyalinosis was graded from 0 to 3 by the method of Takazakura et al. [7, 17, 18, 20]. The severity of arteriosclerosis was graded from 0 to 2 according to the pathological classification by the Renal Pathology Society [6–12, 17, 18]. Four nephrologists evaluated the renal tissue specimens.

### Outcomes

The outcomes of this study were the first occurrence of renal composite events (requirement for dialysis or a 50 % decline in eGFR from baseline) and all-cause mortality. None of the patients received renal transplantation during follow-up. The patients were followed up until the end of 2019 or until death.

### Statistical analysis

Baseline clinical and pathological findings of patients were summarized according to baseline tertile of Hb levels and IFTA status. We categorized mild- and severe IFTA, defined as score ≤ 1 and ≥ 2, respectively. Data were presented as mean and standard deviation for continuous variables and percentage for categorical variables. Mann–Whitney U tests, Kruskal–Wallis tests, and Chi-squared tests were used to identify differences in continuous and categorical variables. Univariate and multivariate linear regression analyses and logistic regression analyses were used to assess the cross-sectional relationship between baseline Hb levels and pathological covariates. For multivariate regression analysis, the pathological covariates were forced into the model. The cumulative incidences of renal composite events and all-cause mortality were evaluated using Kaplan–Meier survival curve. The hazard ratio (HR) and their 95 % confidence intervals (CIs) of different groups stratified by baseline Hb levels and IFTA status on renal composite events and all-cause mortality were calculated using multivariate Cox proportional hazards model analysis. For multivariate Cox regression analysis, we ran three models: Model 1, adjusted for age, gender, eGFR, and presence of albuminuria (proteinuria), Model 2, adjusted for age, gender, eGFR, presence of albuminuria (proteinuria), HbA1c, and systolic blood pressure, and Model 3, adjusted for age, gender, eGFR, presence of albuminuria (proteinuria), HbA1c, systolic blood pressure, and pathological covariates excluding IFTA (i.e. diffuse lesion, nodular lesion, exudative lesion, mesangiolysis, interstitial cell infiltration, arteriolar hyalinosis, arteriosclerosis). These covariates were incorporated in the stepwise procedure. We also evaluated the incremental prognostic value for renal composite events and all-cause mortality associated with the addition of Hb levels to the model that contained 6-clinical variables (age, gender, eGFR, presence of albuminuria [proteinuria], HbA1c, and systolic blood pressure) and presence of severe IFTA using Uno’s C statistics, net reclassification improvement (NRI), and integrated discrimination improvement (IDI). All analyses were performed using SPSS version 24 (SPSS Inc., Chicago, IL, USA) and R 4.0.5. Statistical significance was defined as *p*-values of less than 0.05.

## Results

### Baseline clinical and pathological findings according to Hb levels and IFTA

A total of 233 patients were enrolled in this study, including 79 women and 154 men with a mean age of 58.0 years. Table 1 presents baseline clinical and pathological characteristics of the study population stratified by tertiles according to baseline Hb levels (i.e., ≤ 10.7 in the lowest tertile, 10.8–13.2 in the middle tertile, and ≥ 13.3 in the highest tertile) and IFTA status. There were no patients who have been received erythropoiesis-stimulating agents at the time of renal biopsy. At baseline, 18, 64, 77, and 74 patients were classified into renal IFTA scores 0, 1, 2, and 3, respectively. Age, gender, serum creatinine, eGFR, prevalence of eGFR < 60 mL/min/1.73m^2^, prevalence of micro/macroalbuminuria (mild/severe proteinuria), prevalence of macroalbuminuria (severe proteinuria), HbA1c, systolic blood pressure, IFTA score, diffuse lesion score, prevalence of nodular lesion, prevalence of exudative lesion, prevalence of mesangiolysis, arteriolar hyalinosis score, and arteriosclerosis score were significantly different between the three subgroups of Hb levels. Hemoglobin, age, serum creatinine, eGFR, prevalence of eGFR < 60 mL/min/1.73m^2^, prevalence of macroalbuminuria (severe proteinuria), systolic blood pressure, diffuse lesion score, prevalence of nodular lesion, prevalence of exudative lesion, prevalence of mesangiolysis, interstitial cell infiltration score, arteriolar hyalinosis score, and arteriosclerosis score were significantly different between mild IFTA (score ≤ 1) and severe IFTA (score ≥ 2).

**Table 1:** Baseline clinical and pathological characteristics according to Hb levels and IFTA status

	All	Baseline Hb (g/dL)				IFTA (score)			Mild IFTA				Severe IFTA			
		Lowest tertile	Middle tertile	Highest tertile	*p*	Mild	Severe	*p*	Lowest tertile of Hb	Middle tertile of Hb	Highest tertile of Hb	*p*	Lowest tertile of Hb	Middle tertile of Hb	Highest tertile of Hb	*p*
		≤10.7	10.8–13.2	≥13.3		≤1	≥2									
n	233	76	78	79		82	151		15	26	41		61	52	38	
Clinical covariates																
Hb (g/dL), mean (SD)	12.1	9.4	12.0	14.7	<0.01	13.0	11.6	<0.01	9.4	12.2	14.8	<0.01	9.4	12.0	14.6	<0.01
	(2.4)	(1.1)	(0.7)	(1.1)		(2.3)	(2.3)		(1.5)	(0.6)	(1.2)		(1.1)	(0.7)	(0.9)	
Age (years), mean (SD)	58.0	61.4	59.2	53.5	<0.01	55.2	59.5	<0.05	60.9	59.0	50.6	<0.05	61.5	59.4	56.5	<0.05
	(11.5)	(9.3)	(11.4)	(12.1)		(14.3)	(9.3)		(9.0)	(14.2)	(14.7)		(9.4)	(9.9)	(7.6)	
Male	66.1	61.8	56.4	79.7	<0.01	63.4	67.5	0.52	66.7	50.0	70.7	0.22	60.7	59.6	89.5	<0.01
Serum creatinine (mg/dL), mean (SD)	1.4	2.2	1.3	0.9	<0.01	0.9	1.7	<0.01	1.3	0.9	0.8	<0.01	2.4	1.4	1.0	<0.01
	(1.3)	(1.8)	(1.0)	(0.3)		(0.4)	(1.6)		(0.5)	(0.4)	(0.3)		(1.9)	(1.2)	(0.4)	
eGFR (mL/min/1.73m^2^), mean (SD)	56.8	35.6	57.5	76.4	<0.01	74.0	47.4	<0.01	47.2	70.2	86.0	<0.01	32.8	51.1	66.1	<0.01
	(30.8)	(21.2)	(28.0)	(28.2)		(32.7)	(25.4)		(21.8)	(34.1)	(29.1)		(20.2)	(22.1)	(23.3)	
eGFR <60 mL/min/1.73m^2^	60.5	86.8	61.5	34.2	<0.01	41.5	70.9	<0.01	80.0	53.8	19.5	<0.01	88.5	65.4	50.0	<0.01
Micro/macroalbuminuria or mild/severe proteinuria	85.0	96.1	89.7	69.6	<0.01	81.7	86.8	0.30	86.7	92.3	73.2	0.12	98.4	88.5	65.8	<0.01
Macroalbuminuria or severe proteinuria	63.1	89.5	61.5	39.2	<0.01	41.5	74.8	<0.01	73.3	42.3	29.3	<0.05	93.4	71.2	50.0	<0.01
HbA1c (%), mean (SD)	7.7	6.5	8.3	8.2	<0.01	7.8	7.7	0.52	6.2	8.4	7.9	<0.01	6.6	8.2	8.5	<0.01
	(2.2)	(1.7)	(2.2)	(2.3)		(2.1)	(2.3)		(1.1)	(2.2)	(2.1)		(1.8)	(2.3)	(2.4)	
Systolic blood pressure (mmHg), mean (SD)	140.5	148.8	139.0	134.1	<0.01	131.7	145.4	<0.01	140.4	129.0	130.3	0.15	150.8	144.0	138.2	<0.05
	(21.6)	(22.7)	(20.8)	(18.8)		(20.0)	(21.0)		(22.3)	(18.6)	(19.5)		(22.4)	(20.2)	(17.2)	
Diastolic blood pressure (mmHg), mean (SD)	76.6	77.0	75.3	77.4	0.62	74.8	77.6	0.07	75.6	71.0	76.9	0.21	77.3	77.5	78.1	0.96
	(12.4)	(12.4)	(13.0)	(11.7)		(13.5)	(11.6)		(18.9)	(10.2)	(12.7)		(10.5)	(13.7)	(10.6)	
Total cholesterol (mg/dL), mean (SD)	216.3	223.0	224.5	201.9	0.47	206.6	221.7	0.45	248.7	197.6	196.9	0.36	216.6	238.5	207.3	0.65
	(83.7)	(94.1)	(98.3)	(50.3)		(60.7)	(94.0)		(99.1)	(44.6)	(44.4)		(92.5)	(114.9)	(56.2)	
Body mass index (kg/m^2^), mean (SD)	23.3	22.9	23.0	24.1	0.20	23.5	23.3	0.99	21.3	22.9	24.7	<0.05	23.3	23.1	23.4	0.77
	(3.7)	(3.4)	(3.1)	(4.3)		(4.3)	(3.3)		(2.8)	(3.2)	(4.9)		(3.5)	(3.1)	(3.4)	
Pathological covariates																
IFTA (0–3), mean (SD)	1.9	2.2	1.9	1.5	<0.01	0.8	2.5	<0.01	0.9	0.9	0.7	0.09	2.6	2.5	2.4	0.37
	(0.9)	(0.8)	(0.9)	(1.0)		(0.4)	(0.5)		(0.3)	(0.4)	(0.5)		(0.5)	(0.5)	(0.5)	
Diffuse lesion (0–4), mean (SD)	2.2	2.6	2.3	1.7	<0.01	1.6	2.4	<0.01	2.3	1.7	1.4	<0.01	2.7	2.5	2.0	<0.01
	(0.9)	(0.6)	(1.0)	(0.9)		(0.8)	(0.9)		(0.7)	(0.9)	(0.6)		(0.6)	(1.0)	(1.0)	
Nodular lesion	48.5	68.5	53.2	25.3	<0.01	30.5	58.5	<0.01	46.7	42.3	17.1	<0.05	74.1	58.8	34.2	<0.01
Exudative lesion	34.1	52.1	33.8	17.7	<0.01	19.5	42.4	<0.01	46.7	19.2	9.8	<0.01	53.4	41.2	26.3	<0.05
Mesangiolysis	27.2	39.7	29.9	12.8	<0.01	16.0	33.6	<0.01	20.0	23.1	9.8	0.31	44.8	33.3	16.2	<0.05
Interstitial cell infiltration (0–2), mean (SD)	1.1	1.2	1.2	1.0	0.35	0.8	1.3	<0.01	0.9	1.0	0.7	0.11	1.2	1.3	1.4	0.19
	(0.5)	(0.4)	(0.5)	(0.7)		(0.6)	(0.5)		(0.3)	(0.5)	(0.6)		(0.4)	(0.4)	(0.5)	
Arteriolar hyalinosis (0–3), mean (SD)	2.0	2.2	2.1	1.7	<0.01	1.5	2.3	<0.01	1.7	1.8	1.2	<0.05	2.3	2.3	2.2	0.77
	(1.0)	(0.9)	(0.9)	(1.1)		(1.0)	(0.8)		(1.0)	(0.9)	(1.0)		(0.8)	(0.8)	(0.9)	
Arteriosclerosis (0–2), mean (SD)	1.3	1.5	1.4	1.1	<0.01	1.1	1.4	<0.05	1.4	1.4	0.9	0.05	1.5	1.5	1.3	0.11
	(0.7)	(0.5)	(0.6)	(0.7)		(0.8)	(0.6)		(0.5)	(0.6)	(0.8)		(0.5)	(0.6)	(0.6)	

Data are mean (SD) or percent. Abbreviations: *eGFR* estimated glomerular filtration rate; *Hb* hemoglobin; *IFTA* interstitial fibrosis and tubular atrophy

### Baseline clinical and pathological findings according to Hb levels by IFTA status

We divided the patients into six groups according to baseline Hb levels and IFTA status as follows: the lowest tertile of Hb and mild IFTA (n = 15); the middle tertile of Hb and mild IFTA (n = 26); the highest tertile of Hb and mild IFTA (n = 41); the lowest tertile of Hb and severe IFTA (n = 61); the middle tertile of Hb and severe IFTA (n = 52); and the highest tertile of Hb and severe IFTA (n = 38). The proportion of patients with severe IFTA was 80.3 % (61 of 76) among the lowest tertile of Hb, 66.7 % (52 of 78) among the middle tertile of Hb, and 48.1 % (38 of 79) among the highest tertile of Hb. Baseline clinical and pathological characteristics according to Hb levels by IFTA status are also displayed in Table 1. Clinical and pathological covariates associated with lower Hb levels regardless of IFTA status were older age, higher serum creatinine, lower eGFR, higher prevalence of eGFR < 60 mL/min/1.73m^2^, higher prevalence of macroalbuminuria (severe proteinuria), lower HbA1c, higher diffuse lesion score, higher prevalence of nodular lesion, and higher prevalence of exudative lesion. Lower BMI and higher arteriolar hyalinosis score were associated with lower Hb levels only in mild IFTA. Lower prevalence of male, higher prevalence of micro/macroalbuminuria (mild/severe proteinuria), higher systolic blood pressure, and higher prevalence of mesangiolysis were associated with lower Hb levels only in severe IFTA.

### Associations between baseline Hb levels and pathological covariates

To identify the pathological covariates associated with lower Hb levels, we used univariate and multivariate linear regression analyses (Table 2). In the univariate linear regression analysis, IFTA (R = − 0.327, *p* < 0.01), diffuse lesion (R = − 0.396, *p* < 0.01), nodular lesion (R = − 0.366, *p* < 0.01), exudative lesion (R = − 0.293, *p* < 0.01), mesangiolysis (R = − 0.261, *p* < 0.01), arteriolar hyalinosis (R = − 0.225, *p* < 0.01), and arteriosclerosis (R = − 0.229, *p* < 0.01) negatively correlated with Hb level. In the multivariate linear regression analysis, IFTA was identified as only significant independent predictor of Hb level (standardized coefficient *β* − 0.165, 95 % CI − 0.812 to − 0.007, *p* < 0.05). Univariate and multivariate logistic regression analyses were also performed to define the pathological covariates associated with the lowest tertile of Hb (Table 2). In the univariate logistic regression analysis, IFTA (odds ratio per score increase 1.89, 95 % CI 1.36 to 2.62, *p* < 0.01), diffuse lesion (odds ratio per score increase 2.26, 95 % CI 1.59 to 3.22, *p* < 0.01), presence of nodular lesion (odds ratio 3.39, 95 % CI 1.88 to 6.10, *p* < 0.01), presence of exudative lesion (odds ratio 3.15, 95 % CI 1.76 to 5.64, *p* < 0.01), presence of mesangiolysis (odds ratio 2.44, 95 % CI 1.33 to 4.47, *p* < 0.01), arteriolar hyalinosis (odds ratio per score increase 1.40, 95 % CI 1.04 to 1.89, *p* < 0.05), and arteriosclerosis (odds ratio per score increase 1.64, 95 % CI 1.04 to 2.59, *p* < 0.05) were associated with the lowest tertile of Hb. In the multivariate logistic regression analysis, IFTA remained significantly associated with the lowest tertile of Hb (odds ratio per score increase 1.58, 95 % CI 1.01 to 2.47, *p* < 0.05).

**Table 2:** Pathological covariates identified by regression analyses associated with baseline Hb levels

	Linear regression analyses associated with Hb levels									Binary logistic regression analyses associated with the lowest tertile of Hb													
	Univariate		Multivariate							Univariate							Multivariate						
Pathological covariates	R	*p*	Standardized coefficient B	(95 % CI)					*p*	Odds ratio	(95 % CI)					*p*	Odds ratio	(95 % CI)					*p*
IFTA (score + 1)	–0.327	< 0.01	–0.165	(	–0.812	,	–0.007	)	< 0.05	1.89	(	1.36	,	2.62	)	< 0.01	1.58	(	1.01	,	2.47	)	< 0.05
Diffuse lesion (score + 1)	–0.396	< 0.01	–0.172	(	–0.863	,	0.006	)	0.05	2.26	(	1.59	,	3.22	)	< 0.01	1.58	(	0.95	,	2.62	)	0.08
Presence of nodular lesion	–0.366	< 0.01	-0.141	(	–1.47	,	0.137	)	0.10	3.39	(	1.88	,	6.10	)	< 0.01	1.53	(	0.66	,	3.57	)	0.32
Presence of exudative lesion	–0.293	< 0.01	–0.095	(	–1.184	,	0.249	)	0.20	3.15	(	1.76	,	5.64	)	< 0.01	1.78	(	0.86	,	3.69	)	0.12
Presence of mesangiolysis	–0.261	< 0.01	–0.058	(	–1.09	,	0.472	)	0.44	2.44	(	1.33	,	4.47	)	< 0.01	1.07	(	0.49	,	2.34	)	0.87
Interstitial cell infiltration (score + 1)	–0.123	0.06	–0.016	(	–0.687	,	0.547	)	0.82	1.31	(	0.78	,	2.20	)	0.30	0.96	(	0.45	,	2.01	)	0.91
Arteriolar hyalinosis (score + 1)	–0.225	< 0.01	0.036	(	–0.263	,	0.436	)	0.63	1.40	(	1.04	,	1.89	)	< 0.05	0.84	(	0.56	,	1.24	)	0.38
Arteriosclerosis (score + 1)	–0.229	< 0.01	–0.124	(	–0.917	,	0.014	)	0.06	1.64	(	1.04	,	2.59	)	< 0.05	1.42	(	0.82	,	2.48	)	0.22

Abbreviations: *CI* confidence interval; *Hb* hemoglobin; *IFTA* interstitial fibrosis and tubular atrophy

### Prognosis of renal events and mortality according to the tertile of baseline Hb levels by IFTA status

**Fig. 1:**
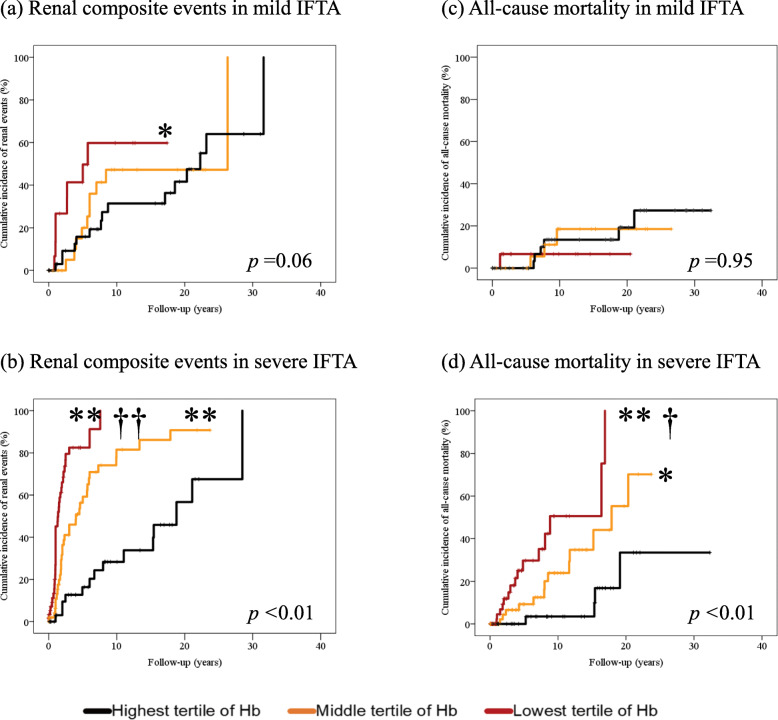
Cumulative incidence of the outcomes according to the tertile of baseline Hb by IFTA status. (**a**) Cumulative incidence of renal composite events stratified by baseline Hb in mild IFTA. Black line, the highest tertile of Hb and mild IFTA group (n = 41), orange line, the middle tertile of Hb and mild IFTA group (n = 26), red line, the lowest tertile of Hb and mild IFTA group (n = 15). *p < 0.05 vs. the highest tertile of Hb. (**b**) Cumulative incidence of renal composite events stratified by baseline Hb in severe IFTA. Black line, the highest tertile of Hb and severe IFTA group (n = 38), orange line, the middle tertile of Hb and severe IFTA group (n = 52), red line, the lowest tertile of Hb and severe IFTA group (n = 61). **p < 0.01 vs. the highest tertile of Hb, and ††*p* < 0.01 vs. the middle tertile of Hb. (**c**) Cumulative incidence of all-cause mortality stratified by baseline Hb in mild IFTA. Black line, the highest tertile of Hb and mild IFTA group (n = 41), orange line, the middle tertile of Hb and mild IFTA group (n = 26), red line, the lowest tertile of Hb and mild IFTA group (n = 15). (**d**) Cumulative incidene of all-cause mortality stratified baseline Hb in severe IFTA. Black line, the highest tertile of Hb and severe IFTA group (n = 38), orange line, the middle tertile of Hb and severe IFTA group (n = 52), red line, the lowest tertile of Hb and severe IFTA group (n = 61). *p < 0.05 vs. the highest tertile of Hb, **p < 0.01 vs. the highest tertile of Hb, and †*p* < 0.05 vs. the middle tertile of Hb. Abbreviations: Hb, hemoglobin; IFTA, interstitial fibrosis and tubular atrophy

The mean follow-up duration was 8.6 years (median = 6.7 years; maximum = 32.4 years) during 1985–2019. There was a total of 119 renal composite events and 42 deaths. Figure 1 shows the cumulative incidence of the outcomes according to the tertile of baseline Hb levels by IFTA status. The cumulative incidences of renal composite events in mild IFTA were not significantly different among the three subgroups of Hb levels, although the cumulative incidence of renal composite events in the lowest tertile of Hb was significantly higher than that of the highest tertile of Hb (Fig. 1a). The cumulative incidences of renal composite events in severe IFTA were significantly different among the three subgroups of Hb levels (Fig. 1b). The cumulative incidence of renal composite events in the lowest tertile of Hb was significantly higher than that of the middle tertile of Hb or that of the highest tertile of Hb in severe IFTA (Fig. 1b). The cumulative incidence of renal composite events in the middle tertile of Hb was also significantly higher than that of the highest tertile of Hb in severe IFTA (Fig. 1b). The cumulative incidences of all-cause mortality in mild IFTA were not significantly different among the three subgroups of Hb levels (Fig. 1c). The cumulative incidences of all-cause mortality in severe IFTA were significantly different among the three subgroups of Hb levels (Fig. 1d). The cumulative incidence of all-cause mortality in the lowest tertile of Hb was significantly higher than that of the middle tertile of Hb or that of the highest tertile of Hb in severe IFTA (Fig. 1d). The cumulative incidence of all-cause mortality in the middle tertile of Hb was also significantly higher than that of the highest tertile of Hb in severe IFTA (Fig. 1d).

### Risks of renal events and mortality according to the tertile of baseline Hb levels by IFTA status

**Fig. 2:**
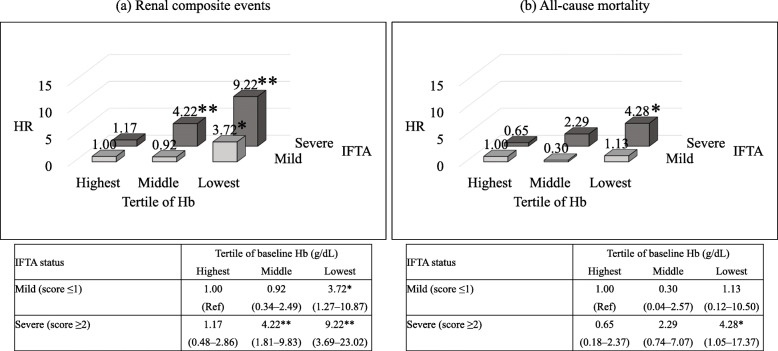
Combined influence of baseline Hb levels and IFTA status on the risks of the outcomes. (a) Multivariable-adjusted HR and 95 % CI for the development of renal composite events. *p < 0.05 vs. the highest tertile of Hb and mild IFTA group. **p < 0.01 vs. the highest tertile of Hb and mild IFTA group. (b) Multivariable-adjusted HR and 95 % CI for the development of all-cause mortality. *p < 0.05 vs. the highest tertile of Hb and mild IFTA group. Adjusted for baseline clinical covariates (age, gender, estimated glomerular filtration rate, presence of albuminuria [proteinuria], HbA1c, and systolic blood pressure) and pathological covariates excluding IFTA. Abbreviations: Hb, hemoglobin; HR, hazard ratio; IFTA, interstitial fibrosis and tubular atrophy

Table 3 shows the adjusted HRs for the outcomes according to the tertile of baseline Hb levels by IFTA status after adjustment for baseline clinical and pathological covariates excluding IFTA. The highest tertile of Hb in each IFTA status was served as the reference group. The risks of renal composite events were significantly higher in the middle tertile of Hb (fully adjusted HR [Model 3 in Table 3] 5.40, 95 % CI 2.39 to 12.18) and in the lowest tertile of Hb (fully adjusted HR, 11.71, 95 % CI 4.84 to 28.31) among severe IFTA, whereas the risk was significantly higher only in the lowest tertile of Hb (fully adjusted HR 6.21, 95 % CI 1.99 to 19.40) among mild IFTA. The risks of all-cause mortality were significantly higher in the middle tertile of Hb (fully adjusted HR 4.47, 95 % CI 1.16 to 17.22) and in the lowest tertile of Hb (fully adjusted HR 8.85, 95 % CI 1.78 to 44.15) among severe IFTA, but not among mild IFTA. Figure 2 shows the combined influence of baseline Hb levels and IFTA status on the risks of the outcomes relative to the group of the highest tertile of Hb and mild IFTA for other groups based on multivariable Cox regression analysis after adjustment for baseline clinical covariates (age, gender, eGFR, presence of albuminuria [proteinuria], HbA1c, and systolic blood pressure) and pathological covariates excluding IFTA. Patients with the highest tertile of Hb and mild IFTA were served as the reference group. The risks of renal composite events became significantly higher in the middle tertile of Hb (adjusted HR 4.22, 95 % CI 1.81 to 9.83) and in the lowest tertile of Hb (adjusted HR 9.22, 95 % CI 3.69 to 23.02) among severe IFTA, whereas the risk became significantly higher in the lowest tertile of Hb (adjusted HR 3.72, 95 % CI 1.27 to 10.87) among mild IFTA (Fig. 2a). The risk of all-cause mortality became significantly higher in the lowest tertile of Hb among severe IFTA (adjusted HR 4.28, 95 % CI 1.05 to 17.37), but not among mild IFTA (Fig. 2b). There were significant interactions of tertile of Hb and IFTA in renal composite events (*p* interaction = 0.04) and all-cause mortality (*p* interaction = 0.04).

**Table 3:** Adjusted HR for the outcomes according to the tertile of baseline Hb by IFTA status

	Mild IFTA (score ≤ 1)			Severe IFTA (score ≥ 2)		
	Highest tertile of Hb	Middle tertile of Hb	Lowest tertile of Hb	Highest tertile of Hb	Middle tertile of Hb	Lowest tertile of Hb
	≥ 13.3	10.8–13.2	≤ 10.7	≥ 13.3	10.8–13.2	≤ 10.7
Participants, n	41	26	15	38	52	61
Renal composite events						
Events, n (%)	15 (36.6)	10 (38.5)	8 (53.3)	14 (36.8)	34 (65.4)	38 (62.3)
Model 1	1 (reference)	0.95 (0.42–2.18)	2.64 (1.06–6.57)*	1 (reference)	3.19 (1.63–6.25)**	7.57 (3.49–16.41)**
Model 2	1 (reference)	0.87 (0.37–2.04)	2.93 (1.17–7.30)*	1 (reference)	3.17 (1.61–6.22)**	7.09 (3.23–15.54)**
Model 3	1 (reference)	0.38 (0.12–1.20)	6.21 (1.99–19.40)**	1 (reference)	5.40 (2.39–12.18)**	11.71 (4.84–28.31)**
All-cause mortality						
Events, n (%)	6 (14.6)	3 (11.5)	1 (6.7)	4 (10.5)	13 (25.0)	15 (24.6)
Model 1	1 (reference)	0.67 (0.17–2.71)	0.52 (0.06–4.47)	1 (reference)	2.75 (0.87–8.75)	8.93 (2.68–29.74)**
Model 2	1 (reference)	0.53 (0.11–2.65)	0.55 (0.06–4.79)	1 (reference)	2.78 (0.88–8.84)	8.66 (2.59–29.03)**
Model 3	1 (reference)	0.09 (0.002–3.65)	0.70 (0.02–29.74)	1 (reference)	4.47 (1.16–17.22)*	8.85 (1.78–44.15)**

Model 1: Adjusted for age, gender, estimated glomerular filtration rate, and presence of albuminuria [proteinuria]. Model 2: Further adjusted for HbA1c and systolic blood pressure. Model 3: Further adjusted for pathological covariates excluding IFTA. *p < 0.05 vs. the highest tertile of Hb. **p < 0.01 vs. the highest tertile of Hb. Abbreviations: *Hb* hemoglobin; *IFTA* interstitial fibrosis and tubular atrophy

We also evaluated the incremental prognostic value of Hb levels over the model that included 6-clinical variables (age, gender, eGFR, presence of albuminuria [proteinuria], HbA1c, and systolic blood pressure) and presence of severe IFTA using Uno’s C-statistics, NRI, and IDI for renal composite events and all-cause mortality. For predicting renal composite events, the addition of Hb levels on the model including severe IFTA was associated with significant improvements in the C-statistics value (0.745 versus 0.789, delta C statistics = 0.043 [95 %CI 0.006 to 0.081]), the NRI statistics (10-year risk prediction: 0.401 [95 %CI 0.196 to 0.582], *p* < 0.01), and the IDI statistics (10-year risk prediction: 0.079 [95 %CI 0.022 to 0.144], *p* < 0.01). For predicting all-cause mortality, we could not find significant improvements in the C-statistics value (0.754 versus 0.757, delta C statistics = 0.003 [95 %CI -0.003 to 0.031]), the NRI statistics (10-year risk prediction: 0.110 [95 %CI -0.188 to 0.319], *p* = 0.52), and the IDI statistics (10-year risk prediction: 0.011 [95 %CI -0.007 to 0.080], *p* = 0.25).

## Discussion

The present study showed two findings: (i) IFTA was an independent pathological factor associated with lower Hb levels in multivariate analysis, and (ii) the effect of lower Hb levels on renal composite events and all-cause mortality were greater in patients with severe IFTA than in those with mild IFTA.

First, we showed that IFTA was an independent pathological predictor of lower Hb levels by multivariate regression model. Although we observed multiple correlates of pathological covariates for baseline Hb levels, the severity of IFTA was particularly strongly associated with baseline low Hb levels. A previous study focusing on diabetic nephropathy demonstrated that advanced IFTA was associated with both baseline Hb and decrease in Hb during the follow-up period [3]. Notably, we showed that impacts of this relationship on the outcomes in patients with type 2 diabetes and biopsy-proven diabetic nephropathy. There are plausible mechanisms linking IFTA to the progression or renal anemia. In renal fibrosis, the reduced or even loss of the ability of activated fibroblasts to produce erythropoietin is a major cause of renal anemia [21, 22]. In turn, anemia may cause renal hypoxia, leading to renal fibrosis [1, 21, 22]. These clinical and experimental findings suggest that renal anemia and renal fibrosis have a causal relationship with one another in CKD.

Next, we showed that the impacts of lower Hb levels on renal composite events and all-cause mortality were greater in severe IFTA than in mild IFTA. Previous studies showed that lower Hb levels in diabetic nephropathy/DKD were associated with adverse clinical outcomes [1, 2]. However, most of these studies did not consider the severity of renal pathology. Our study provided a more detailed analysis of both the severity of anemia and renal pathological findings in diabetic nephropathy. The cumulative incidence showed an association between lower Hb levels and greater increase of renal composite events and all-cause mortality in patients with severe IFTA, but not in those with mild IFTA. The risk of renal composite events increased from the highest tertile of Hb to the middle tertile of Hb, to the lowest tertile of Hb in patients with severe IFTA, even after adjusting for baseline clinical and pathological covariates. The risk of all-cause mortality increased only in patients with severe IFTA. There were significant interactions of tertile of Hb and IFTA in renal composite events and all-cause mortality.

Regarding the incremental prognostic value of adding Hb levels to the model including severe IFTA, we demonstrated significant improvements of the values in the C statistics, NRI, and IDI for renal composite events. Our results might suggest the usefulness of more intensive control of anemia in patients with severe IFTA than in those with mild IFTA to improve renal prognosis in type 2 diabetes with diabetic nephropathy.

The strength of the present study is that we directly evaluated IFTA status using renal biopsy. However, pathological evaluation is uncommonly used in diabetic nephropathy with a typical clinical course. Recent studies showed that higher degrees of renal IF assessed by functional magnetic resonance imaging were associated with a faster eGFR decline in CKD [23, 24]. Alternatively, the result of the first multicenter, multiparametric, functional magnetic resonance imaging study in 122 participants with stage 3b–4 CKD over the course of 1 year was not in line with those of previous single-center studies [25]. Larger trials with longer follow-up would be required to confirm the surrogate markers associated with kidney lesions in diabetic nephropathy.

There were several limitations in our study. First, there may have been an influence of bias from limiting the study to patients who underwent renal biopsy. Second, only a single measurement of Hb at baseline might have caused the misclassification of some study patients. Third, we did not have data on serum erythropoietin levels. Although 11.5 % of the lowest tertile of Hb and 24.6 % of the middle tertile of Hb in patients with severe IFTA showed preserved eGFR (≥ 60 mL/min/1.73 m^2^) in the present study, one previous study demonstrated that low erythropoietin levels predicted rapid eGFR decline in type 2 diabetic patients with anemia [26]. Fourth, we did not consider the use of erythropoiesis-stimulating agents during the follow-up period. As to treatment contents, recent studies have revealed that hypoxia-inducible factor-prolyl hydroxylase inhibitors might suppress tubulointerstitial fibrosis by suppressing the transformation of renal interstitial fibroblasts in addition to enhancing erythropoiesis [27, 28]. Furthermore, some previous studies revealed that sodium-glucose cotransporter 2 inhibitors might improve tubulointerstitial hypoxia, allowing fibroblasts to resume erythropoietin production [29, 30]. On this context, future studies focusing on the relationship between diabetic kidney lesions and these treatment contents are needed. Fifth, the data on urinary protein excretion were used to classify the category of albuminuria in the absence of the data on urinary albumin excretion. Finally, due to the retrospective nature of this study, we could not fully investigate the causes of anemia and death in some cases. Although iron and erythropoietin deficiencies and hyporesponsiveness to the actions of erythropoietin are the major causes of anemia in CKD patients, anemia in diabetic patients with CKD result from one or more mechanisms [31, 32]. In our study, lower BMI and higher arteriolar hyalinosis score were associated with lower Hb levels only in mild IFTA. Although the underlying mechanism is unknown, one previous study of biopsy-proven diabetic nephropathy has also reported consistent results [3]. Nevertheless, long-term observation of 233 patients with type 2 diabetes and biopsy-proven diabetic nephropathy is important for understanding the predictive effect of kidney lesions on clinical outcomes.

## Conclusions

This retrospective analysis showed that lower Hb levels were associated with greater risks of renal events and mortality in patients with severe IFTA than in those with mild IFTA. Our results support the importance of managing anemia in patients with diabetic nephropathy, particularly associated with severe IFTA.

## Data Availability

The inspection and usage of the data in this study is restricted according to the ethical approval. All data relevant to the study are included in the article.

## Abbreviations

CI: Confidence intervals
CKD: Chronic kidney disease
DKD: Diabetic kidney disease
eGFR: Estimated glomerular filtration rate
Hb: Hemoglobin
HR: Hazard ratio
IDI: Integrated discrimination improvement
IFTA: Interstitial fibrosis and tubular atrophy
NRI: Net reclassification improvement
